# Sepsis affects kidney graft function and one-year mortality of the recipients in contrast with systemic inflammatory response

**DOI:** 10.3389/fmed.2022.923524

**Published:** 2022-07-29

**Authors:** Marek Protus, Eva Uchytilova, Veronika Indrova, Jan Lelito, Ondrej Viklicky, Petra Hruba, Eva Kieslichova

**Affiliations:** ^1^Department of Anesthesiology, Resuscitation and Intensive Care, Institute for Clinical and Experimental Medicine, Prague, Czechia; ^2^First Faculty of Medicine, Charles University, Prague, Czechia; ^3^Department of Nephrology, Transplant Centre, Institute for Clinical and Experimental Medicine, Prague, Czechia; ^4^Transplantation Laboratory, Experimental Medicine Centre, Institute for Clinical and Experimental Medicine, Prague, Czechia

**Keywords:** kidney transplantation, sepsis, systemic inflammatory response syndrome, mortality, graft loss

## Abstract

**Background:**

Infections remain a major cause of morbidity and mortality after kidney transplantation. The aim of our study was to determine the effect of sepsis on kidney graft function and recipient mortality.

**Methods:**

A prospective, observational, single-center study was performed. Selected clinical and biochemical parameters were recorded and compared between an experimental group (with sepsis, *n* = 34) and a control group (with systemic inflammatory response syndrome, *n* = 31) comprising kidney allograft recipients.

**Results:**

Sepsis worsened both patient (HR = 14.77, *p* = 0.007) and graft survival (HR = 15.07, *p* = 0.007). Overall one-year mortality was associated with age (HR = 1.08, *p* = 0.048), APACHE II score (HR = 1.13, *p* = 0.035), and combination immunosuppression therapy (HR = 0.1, *p* = 0.006), while graft survival was associated with APACHE II (HR = 1.25, *p* = 0.004) and immunosuppression. In sepsis patients, mortality correlated with the maximal dose of noradrenalin (HR = 100.96, *p* = 0.008), fungal infection (HR = 5.64, *p* = 0.024), SAPS II score (HR = 1.06, *p* = 0.033), and mechanical ventilation (HR = 5.97, *p* = 0.033), while graft survival was influenced by renal replacement therapy (HR = 21.16, *p* = 0.005), APACHE II (HR = 1.19, *p* = 0.035), and duration of mechanical ventilation (HR = 1.01, *p* = 0.015).

**Conclusion:**

In contrast with systemic inflammatory response syndrome, septic kidney allograft injury is associated with early graft loss and may represent a significant risk of mortality.

## Introduction

The number of kidney transplantations is steadily increasing (1). Although allograft survival has improved (2), the potential for surgical complications combined with the impact of immunosuppression predisposes recipients to infectious complications (3, 4). In particular, bloodstream infections (BSI) remain a major cause of morbidity, graft dysfunction and mortality after transplantation. When accompanied by septic shock, mortality can reach up to 50% (5).

Predisposing factors include those present in the recipient or donor before transplantation as well as those secondary to intraoperative and post-transplant events (6). Knowledge of the previous and current immunosuppression burden as well as the time course of infectious episodes after transplantation can guide clinicians toward devising the most appropriate treatment. In the first 6 months, infections are usually related to postoperative complications, manipulation of the urinary tract or viral reactivation. Urinary tract infections (UTI) are also the main source of BSI, followed by catheter-related and wound infections (5).

Perioperative antibiotic prophylaxis should be carefully considered based on the epidemiological situation at the transplant center concerned, the possible colonization of the recipient, and other risk factors. Despite a decrease in the incidence of infectious complications due to routine perioperative and long-term prophylaxis, recipients remain at significant risk of developing infections from multidrug-resistant (MDR) pathogens. MDR bacteremia results in significantly poorer clinical outcomes and higher overall case-fatality rates compared with other etiologies (7).

Septic acute kidney injury (AKI) is defined as the acute impairment of function and organ damage linked with long-term adverse outcomes depending on the extent of acute injury superimposed on the underlying organ reserve (8). Early and appropriate antimicrobial therapy along with septic source control is a cornerstone in its prevention (9). Since septic AKI is not characterized by renal hypoperfusion, restricting resuscitation fluid volumes is feasible (10). While the level of renal protection provided by the most commonly used vasopressors is comparable (11), maintaing the target mean arterial pressure is likely more important (12). Ultimately, a proportion of sepsis patients will undergo renal replacement therapy (RRT). Although commencing RRT at an early phase of both sepsis and AKI can prevent fluid overload and organ injury by removing inflammatory mediators, it can also expose patients to a number of adverse effects, including inadequate antibiotic dosing (13).

Immunosuppressants are used in many different combinations after kidney transplantation, depending on the risk of rejection in the individual patient, time course following transplantation, previous adverse effects and local protocols. The risk of rejection, potentiated by a reduction or discontinuation of immunosuppressive therapy during sepsis, should always be balanced against life-threatening septic complications (14).

The primary aim of our study was to determine the influence of sepsis on kidney allograft function and to identify possible risk factors that contribute to the development of septic acute kidney allograft injury. The secondary aim was to evaluate the mortality of sepsis patients in comparison with kidney allograft recipients with systemic inflammatory response syndrome (SIRS).

## Materials and methods

### Study design and patient population

This prospective observational study was performed between 2018 and 2020 at the intensive care unit (ICU) of the Transplant Centre at the Institute for Clinical and Experimental Medicine (IKEM), Prague. Consecutive kidney transplant patients admitted to ICU for management of a first episode of sepsis were prospectively included. Inclusion criteria were as follows: kidney transplant patients with a first episode of sepsis; age ≥18 years; ICU stay ≥24 h. Exclusion criteria were: age <18 years; objection to participating in the study; severe underlying disease with poor prognosis and/or a life expectancy of less than 24 h; clinical history involving loss of a previous organ transplant graft; recent discontinuation of immunosuppression therapy. The absence of any antibiotic treatment within at least 1 month prior to enrollment was conditional. In all experimental patients, the first dose of antibiotics was given after enrollment as part of sepsis therapy. The control group included kidney transplant patients diagnosed with SIRS without infection within the first 30 days after transplantation. Exclusion criteria for the control group were as follows: clinical signs of systemic or local infection within 30 days after transplantation; age < 18 years; objection to participating in the study. In cases involving repeat admissions of a patient (in either group) to ICU, only the first admission was considered.

### Data collection and interventions

Selected clinical and biochemical data were recorded for both groups. Clinical data were derived from the medical records, clinical examinations, and anamneses of patients. Biochemical examinations of serum biochemistry, blood counts, blood coagulation parameters, and laboratory markers of sepsis/SIRS were performed at an accredited laboratory (ISO 15189) at IKEM’s Department of Laboratory Methods. Pathogen detection (fungal and bacterial) was performed using standard microbiological examination procedures, and detection of microbial nucleic acids by polymerase chain reaction (PCR). Acute graft rejection was diagnosed according to specific (oliguria, serum biochemistry abnormalities) or non-specific (generalized malaise, fever, and anorexia) symptoms and confirmed by immunological testing and histology of the graft biopsy. A graft biopsy was performed in all cases of suspected acute graft rejection, with histology evaluated by experienced pathologists from IKEM’s Department of Clinical and Transplant Pathology.

Selected clinical parameters and laboratory markers were recorded in order to estimate possible organ dysfunction and severity using the Acute Physiology and Chronic Health Evaluation II (APACHE II) score, the Simplified Acute Physiology Score (SAPS II) and, in patients with sepsis, the Sepsis-related Organ Failure Assessment score (SOFA). The APACHE severity score was calculated from the worst parameters obtained within the first 24 h after admission to ICU, the SAPS II score was collected within the first 24 h of the ICU stay, and the SOFA score was calculated daily during the ICU stay. The following clinical and laboratory markers were recorded: demographic data, comorbid conditions, prophylaxis, type of immunosuppression, sites and type of infection (community- or hospital-acquired), septic shock development in the sepsis group. Vital signs such as mental status, temperature, hemodynamic and ventilation parameters, urine output and fluid balance were also recorded.

Interventions such as antibiotic use, vasopressor administration (including epinephrine, norepinephrine, and dobutamine), mechanical ventilation (MV), renal replacement therapy (RRT), and nutritional therapy were recorded. Any infectious episodes, acute tubular necrosis or acute rejection of the allograft occurring within 1 year of inclusion were monitored. In the sepsis group, the time from transplantation to inclusion (days), reduction or withdrawal of immunosuppression, and the number of days without immunosuppression were recorded. Sepsis was diagnosed in accordance with the Surviving Sepsis Campaign (SSC) consensus guidelines based on clinical examination, imaging methods, and laboratory testing, including microbiological identification of the infectious agent by microbiological, immunological and molecular-biological techniques. Sepsis treatment was carried out according to standards based on antimicrobial therapy, source control, early goal-directed therapy (EGDT), and supportive treatment (15, 16).

### Immunosuppression

Our standard immunosuppressive protocol consisted of induction agents and a combination of extended-release tacrolimus (Advagraf, Astellas), mammalian target of rapamycin inhibitors (mTORi, Rapamune, Pfizer), mycophenolic acid (Myfortic, Novartis) and prednisone. The standard protocol was adjusted according to individual immunological risk. Episodes of rejection were treated with intravenous steroids or lymphocyte-depleting agents.

Reduction or withdrawal of immunosuppression in sepsis patients was performed according to standard procedures used at our transplant center. Nevertheless, corticosteroid administration was not discontinued in order to allow for septic shock-associated adrenocortical insufficiency. The suitability of sepsis patients to resume immunosuppressive therapy was assessed daily.

### Outcomes

Clinical outcomes of patients, represented by in-hospital mortality, one-year mortality and kidney allograft function, were assessed one year after inclusion in the study. Allograft function was classified as impaired in cases where serum creatinine (stable before septic episode) increased above 150 μmol/l and subsequently failed to return to the preceding value within the defined time period. Allograft function was defined as lost in cases where chronic hemodialysis treatment was reinitiated within 1 year of enrollment. Selected parameters were compared between the group of kidney transplant patients with sepsis and the group of kidney transplant patients with SIRS, including the number and type of consecutive infectious episodes. Hospital-acquired infections were defined as healthcare-associated infections in cases where the first symptoms occurred more than 48 h after admission to hospital. Risk factors for one-year mortality, impaired kidney function and loss of kidney graft function within one year after inclusion were also identified.

### Statistical analysis

Continuous variables were reported as medians and interquartiles with range determination (minimum, maximum). Categorical variables were expressed as *n* and a percentage of the total. Continuous variables were compared using the two-sample Wilcoxon rank-sum test and categorical variabes using Fisher’s exact test. Survival analysis was performed using the Kaplan–Meier method, with differences between groups compared using the log-rank test. Univariable Cox proportional-hazards models were used to estimate hazard ratios (HR) and 95% confidence intervals (CI) of potential risk factors for patient and graft survival. Binary logistic regression was used to estimate odds ratios (OR) and 95% confidence intervals (CI) of potential risk factors for kidney graft dysfunction one year after study enrollment. A *p*-value < 0.05 was considered statistically significant throughout the study. Statistical analysis was performed using R-studio software, version 4.1.3 (2022-03-10) (Development for R. RStudio, Inc., Boston, MA, United States) and JMP 15.2.0, 2019 (SAS Institute, Inc).

## Results

The experimental group consisted of 34 kidney transplant recipients readmitted to hospital because of sepsis; the control group comprised 31 kidney transplant recipients with SIRS only and without clinical or laboratory signs of BSI admitted to ICU immediately after transplantation.

### Demographic and clinical characteristics

In principle, baseline demographic and clinical characteristics did not significantly differ between groups. The median age was 60 years for sepsis patients and 48 years for controls. Males accounted for 47% of patients in the sepsis group and 71% in the control group. The median body mass index was 26.4 for the sepsis group and 25.8 for controls. The APACHE II severity score was significantly lower in controls with a median of 12, while the sepsis group had a median of 19.5 (*p* = 0.001) (Table 1). The median time from transplantation to enrollment was 254 days in the sepsis group (Table 2).

**TABLE 1:** Baseline demographic and clinical characteristics of the study population with a comparison of variables between both groups.

Variable	SIRS group	SIRS group % or range	Sepsis group	Sepsis group % or range	*P*-value
Age (years)	48 (44, 63)	19–78	60 (49.5, 68)	22–82	0.165
Sex (male)	22/31	71%	16/34	47%	0.077
APACHE II	12 (10, 14)	8–26	19.5 (15.8, 25)	7–33	0.001*
BMI	25.8 (23.5, 30.4)	17.9–39.1	26.4 (23.4, 29.0)	20–39	0.564
**Comorbidities**					
Type I diabetes	4/31	13%	6/34	18%	0.736
Type II diabetes	7/31	23%	10/34	29%	0.582
COPD	1/31	3%	4/34	12%	0.358
Cancer	0/31	0%	4/34	12%	0.115
IHD	8/31	26%	18/34	53%	0.042*
Hypertension	31/31	100%	24/34	70%	0.005*
CHF	2/31	6%	0/34	0	0.602
Liver disease	2/31	6%	0/34	0	0.223
**Chronic immunosuppression**					
Tacrolimus	29/31	94%	24/34	71%	0.086
Cyclosporine	2/31	6%	4/34	12%	0.674
MMF	29/31	94%	21/34	62%	0.003*
mTORi	2/31	6%	1/34	3%	1.0
**Type of transplantation**					
1st, 2nd, 3rd kidney	25/3/0	80%/10%/0%	26/2/1	76%/6%/3%	-
Pancreas and kidney	3/31	10%	5/34	15%	-

Data are presented as n (%) or medians and interquartile ranges. SIRS: systemic inflammatory response syndrome, APACHE II, Acute Physiology and Chronic Health Evaluation II; BMI, body mass index; COPD, chronic obstructive pulmonary disease; IHD, ischemic heart disease; CHF, chronic heart failure; MMF, mycophenolate mofetil; mTROi, mammalian target of rapamycin inhibitor. Boldface indicates statistical significance where *p* < 0.05 (*).

**TABLE 2:** Kidney graft function one year after enrollment, time from transplantation to enrollment in days, length of stay, in-hospital mortality, and one-year mortality.

Variable	SIRS group	SIRS group % or range	Sepsis group	Sepsis group % or range	*P*-value
Stable	23/31	74%	13/34	38%	0.006*
Impaired function	8/31	26%	7/34	20%	0.770
Loss of graft function	0/31	0	7/34	20%	0.011*
Hemodialysis	0/31	0	7/34	20%	0.011*
Episode of ATN after enrollment (1 year)	9/31	29%	12/34	35%	0.608
Days from transplantation to enrollment	0	–	254 (50.8, 3333.5)	5–6882	–
Length of stay (days)	13 (10, 14)	7–28	20.5 (12.8, 34)	1–104	0.001*
In-hospital mortality	0/31	0	2/34	6%	0.493
1-year mortality	0/31	0	7/34	20%	0.012*

Data are presented as n (%) or medians and interquartile ranges. SIRS: systemic inflammatory response syndrome, impaired function indicates serum creatinine >150 μmol/l, ATN: acute tubular necrosis. Boldface indicates statistical significance where *p* < 0.05 (*).

Patients from both groups had similar comorbidities, with 70% of sepsis patients and 100% of control patients suffering from hypertension, which was the most common comorbidity in both groups followed by ischemic heart disease. There was a slight diference in some other comorbidities between the two groups. Signs of chronic heart failure were identified in 6% of control patients. The second most frequent disease was type two diabetes, found in 29% of sepsis patients and 23% of controls. Type one diabetes was less common, diagnosed in 18% of sepsis patients and 13% of controls. Cancer was identified in 12% of sepsis patients, with the same percentage of patients from this group displaying chronic obstructive pulmonary disease (COPD). While no patient in the control group had cancer, 3% of controls exhibited COPD. Two control patients had a history of liver disease.

In the majority of cases (71% of sepsis patients, 94% of controls), chronic immunosuppressive therapy administered to kidney recipients was based on tacrolimus, with cyclosporine used in 12% of sepsis patients and 6% of controls. As part of combination immunosuppression treatment, mycophenolate mofetil was given to 62% of sepsis patients, a significantly lower percentage than controls (94%) (*p* = 0.003). Only 3% of sepsis patients and 6% of controls had mTORi (Table 1).

With respect to the type of kidney transplantation, no differences were found between the two groups. Most patients received a first kidney graft, represented by 76% of sepsis patients and 80% of controls. In sepsis patients, only 6% underwent a second kidney transplantation, while 3% underwent a third kidney transplantation. Similarly, in control patients, only 10% underwent a second transplantation, but no patients underwent a third transplantation. The remaining 15% of sepsis patients and 10% of control patients underwent a first simultaneous pancreas and kidney transplantation (Table 1).

### Clinical outcomes

The main aims of our study were to evaluate kidney graft function one year after a sepsis event and then to compare outcomes with graft function in SIRS (control) patients. Graft function remained stable in only 13 sepsis patients (38%) in comparison with 23 control patients (74%) (*p* = 0.006). In 20% of sepsis patients and 26% of controls, graft function was classified as impaired one year after enrollment. An episode of acute tubular necrosis (ATN) occurred within the year in 35% of sepsis patients and 29% of controls, although these differences were not statistically significant. Seven kidney recipients (20%) from the sepsis group lost graft function completely within 1 year after the sepsis event before returning to hemodialysis, whereas in the control group all grafts remained functional to such a degree that no patient required dialysis within the defined time period (*p* = 0.011). A biopsy was performed in suspected cases of allograft rejection within 1 month of the sepsis episode, representing a total of 14 patients from the sepsis group (41%). Acute tubular necrosis was identified in 7 patients and tubular atrophy in 2 patients. Rejection changes were not observed in any of the biopsies.

**FIGURE 1:**
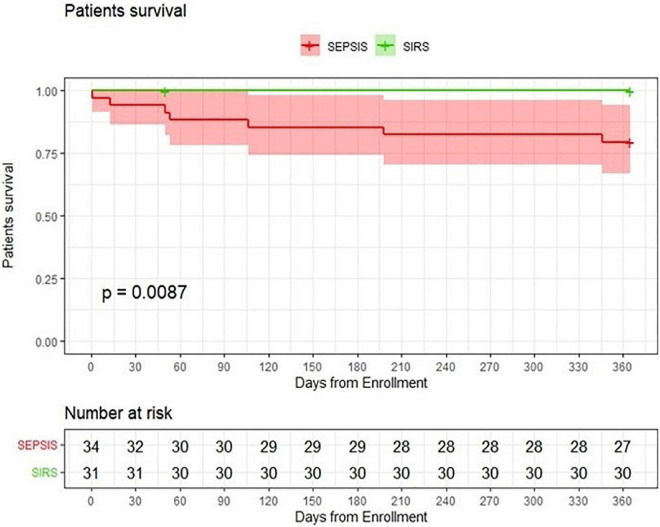
Probability of patients survival. Comparison of kidney transplant patients with sepsis with a group of kidney transplant patients with SIRS.

The median hospital stay in patients with sepsis was 20.5 days, significantly longer than the median hospital stay in control patients (13 days) (*p* = 0.001). In-hospital mortality did not differ significantly between the two groups. However, one-year mortality was higher in the sepsis group (*p* = 0.012) (Table 2) with significantly impaired 1-year survival (logrank *p* = 0.0087) (Figure 1).

### Infectious complications

Sepsis patients also proved more susceptible to infectious complications. Six (18%) developed one complication, while 20 (59%) developed more than one infectious complication within the defined time period. These cases occurred significantly more frequently than controls (*p* = 0.012). Unsurprisingly, the urinary tract was the most common site of infection (65% of sepsis patients and 52% of controls) followed by abdominal and respiratory infections. Three patients from the sepsis group developed a biliary tract infection. In 12 out of 34 patients from the sepsis group, BSI confirmed by positive hemoculture occurred repeatedly. As anticipated, more hospital-acquired than community-acquired infections were recorded within the year in both groups of patients. Overall, the number of infectious episodes of both types was higher in the sepsis group, as mentioned above (Table 3).

**TABLE 3:** Infectious complications within the first year after enrollment.

Variable	SIRS group n/N	SIRS group % or range	Sepsis group n/N	Sepsis group % or range	*P*-value
One sepsis event	8/31	26%	6/34	18%	0.559
More than one sepsis event	8/31	26%	20/34	59%	0.012*
**Site of infection**					
Urinary	16/31	52%	22/34	65%	0.322
Lung	2/31	6%	3/34	9%	1.0
Biliary tract	0	0	3/34	9%	0.240
Abdomen	2/31	6%	7/34	20%	0.153
Positive hemoculture	0		12/34	35%	-
**Type of infection**					
Community-acquired	9/31	29%	13/34	38%	0.600
Hospital-acquired	12/31	39%	20/34	59%	0.138

SIRS, systemic inflammatory response syndrome. Boldface indicates statistical significance where *p* < 0.05 (*).

### Risk factors associated with mortality

Taking kidney graft recipients with sepsis separately, we analyzed possible risk factors associated with increased 1-year mortality. According to univariable Cox regression, the following were significant mortality factors: SAPS II score (HR = 1.06, *p* = 0.033), the presence of fungal infection (HR = 5.64, *p* = 0.024), the need for mechanical ventilation (HR = 5.97, *p* = 0.033), but not duration, and the maximum dose of norepinephrine (HR = 100.96, *p* = 0.008). However, none of the demographic characteristics and comorbidities, APACHE and SOFA scores, the duration of immunosuppression withdrawal due to sepsis, or the site and source of infection played a significant role. Unexpectedly, we found that neither the development of septic shock nor any clinical sign of organ dysfunction due to sepsis, such as acute lung injury (ALI), acute kidney injury requiring renal replacement therapy, lactic acidosis, low platelet count, or elevated serum bilirubin level, significantly affected the one-year mortality of patients (Table 4A).

**TABLE 4A:** Univariable Cox regression analysis of possible risk factors associated with one-year mortality in patients with sepsis (*n* = 34).

Variable	HR (95% CI)	*P*-value
BMI	1.02 (0.89–1.18)	0.750
DM I + II	0.47 (0.09–2.41)	0.363
APACHE II score	1.06 (0.94–1.2)	0.340
SOFA score	1.03 (0.99–1.71)	0.060
SAPS II score	1.06 (1.01–1.12)	0.033*
Days without immunosuppression	1.00 (1.00–1.01)	0.518
Community-acquired	0.31 (0.04–2.56)	0.275
Hospital-acquired	3.25 (0.39–27.04)	0.275
Fungal infection	5.64 (1.25–25.37)	0.024*
MDR bacteria	6.35 (0.76–52.85)	0.087
Viral infection	1.55 (0.19–12.87)	0.686
G- infection	0.65 (0.14–2.89)	0.567
G+ infection	1.13 (0.14–9.39)	0.911
G- and G+ infection	1.58 (0.31–8.18)	0.584
Acute lung injury	2.21 (0.49–9.87)	0.300
Lactic acidosis	5.0 (0.6–41.62)	0.137
Acute kidney injury	0.41 (0.08–2.14)	0.291
Serum bilirubin 20 μmol/l	2.11 (0.47–9.42)	0.329
Thrombocytopoenia	0.39 (0.05–3.23)	0.382
Septic shock	5.00 (0.6–41.62)	0.137
Mechanical ventilation	5.97 (1.15–30.93)	0.033*
Duration of mechanical ventilation	1.01 (1–1.02)	0.209
RRT	1.07 (0.21–5.53)	0.934
Noradrenaline maximum dose	100.96 (3.41–2985.66)	0.008*

APACHE II, Acute Physiology and Chronic Health Evaluation II; BMI, body mass index; DM I + II, diabetes mellitus type I and type II; SOFA, Sequential Organ Failure Assessment score; SAPS II, Simplified Acute Physiology Score II; RRT, renal replacement therapy; HR, hazard ratio; CI, confidence interval; MDR, multidrug-resistant; G-, gram-negative bacteria; G+, gram-positive bacteria. Boldface indicates statistical significance where *p* < 0.05 (*).

Analyzing the data on all kidney graft recipients from both groups together, we found that patients with sepsis had a 14.8-times-worse one-year survival (HR = 14.77, *p* = 0.007). Immunosuppression protocol without MMF (HR = 0.1, *p* = 0.006), APACHE II score (HR = 1.13, *p* = 0.035), and age (HR = 1.08, *p* = 0.048) was associated with 1-year mortality based on univariable Cox regression. On the other hand, comorbidities such as hypertension, both types of diabetes, sex, and BMI did not play a significant role. We found no association between immunosuppression protocol (tacrolimus, cyclosporine, exclusively corticosteroid immunosupression) and one-year mortality (Table 4B).

**TABLE 4B:** Univariable Cox regression analysis of all-cause one-year mortality for the whole patient cohort (*n* = 65).

Variable	HR (95% CI)	*P*-value
Immunosuppression without MMF	0.10 (0.02–0.52)	0.006*
APACHE II	1.13 (1.08–1.26)	0.035*
Age	1.08 (1.00–1.16)	0.048*
Hypertension	0.31 (0.06–1.58)	0.156
Prednisone only	4.35 (0.52–36.23)	0.174
Sex (male)	0.51 (0.12–2.30)	0.384
DM I + II	0.57 (0.11–2.95)	0.503
IHD	0.58 (0.11–2.97)	0.51
Cyclosporine	1.72 (0.21–14.32)	0.615
BMI	1.01 (0.87–1.17)	0.896
Tacrolimus	1.09 (0.13–9.03)	0.938
Sepsis patients	14.77 (1.80–1917.57)	0.007*

APACHE II, Acute Physiology and Chronic Health Evaluation II; BMI, body mass index; DM I + II, diabetes mellitus type I and type II; HR, hazard ratio; CI, confidence interval; SIRS, systemic inflammatory response syndrome; MMF, mycophenolate mofetil; IHD, ischemic heart disease. Boldface indicates statistical significance where *p* < 0.05 (*).

### Risk factors associated with graft dysfunction

Binary logistic regression was used to identify risk factors associated with impairment of graft function in kidney transplant recipients from both groups. Surprisingly, the only significant risk factor linked to impairment of kidney graft function within one year was BMI (*p* = 0.042), whereas age, APACHE II score, the source of infection and recurrent infections within the defined period did not seem to play an important role (Supplementary Table 1).

To identify the risk factors associated with complete loss of graft function in the sepsis group, univariable Cox regression analysis was performed. Based on our results, APACHE II score (HR = 1.19, *p* = 0.035), duration of mechanical ventilation (HR = 1.01, *p* = 0.015), and the need for renal replacement therapy during sepsis (HR = 21.16, *p* = 0.005) were crucial factors. Conversely, other demographic parameters and comorbidities (BMI, age, sex, and diabetes), immunosuppression-free duration, source of infection, type of microorganism, SOFA, SAPS II score, or presence of septic shock requiring vasopressor circulatory support accompanied by ALI, as well as lactic acidosis, elevated serum bilirubin or low platelet count, did not play a significant role (Table 5A).

**TABLE 5A:** Univariable Cox regression of possible risk factors associated with kidney graft failure in the sepsis group one year after enrollment (*n* = 34).

Variable	HR (95%CI)	*P*-value
BMI	1.02 (0.88–1.18)	0.789
Age (years)	1.03 (0.97–1.08)	0.392
Sex (male)	7.78 (0.94–64.74)	0.058
DM I + II	0.98 (0.22–4.39)	0.980
APACHE II score	1.19 (1.01–1.41)	0.035*
SOFA score	1.1 (0.88–1.38)	0.391
SAPS II score	1.02 (0.97–1.07)	0.437
Days without immunosuppression	0.99 (0.96–1.03)	0.631
Community-acquired	0.33 (0.04–2.72)	0.302
Hospital-acquired	3.05 (0.37–25.35)	0.302
Fungal infection	0.97 (0.12–8.03)	0.974
MDR bacteria	0.32 (0.06–1.65)	0.173
G- infection	1.24 (0.24–6.4)	0.797
G+ infection	1.18 (0.14–9.84)	0.877
G- and G+ infection	0.62 (0.08–5.15)	0.658
Acute lung injury	2.13 (0.48–9.55)	0.323
Lactic acidosis	0.91 (0.2–4.05)	0.898
Serum bilirubin 20 μmol/l	0.46 (0.06–3.79)	0.467
Thrombocytopoenia	3.51 (0.79–15.72)	0.1
Septic shock	0.91 (0.2–4.05)	0.898
Mechanical ventilation	3.02 (0.67–13.52)	0.149
Duration of mechanical ventilation	1.01 (1.00–1.02)	0.015*
RRT	21.16 (2.53–177.11)	0.005*
Noradrenaline maximum dose	0.46 (0.02–10.99)	0.633

APACHE II, Acute Physiology and Chronic Health Evaluation II; BMI, body mass index; DM I + II, diabetes mellitus type I and type II; SOFA, Sequential Organ Failure Assessment score; SAPS II, Simplified Acute Physiology Score II; RRT, renal replacement therapy; HR, hazard ratio; CI, confidence interval; MDR, multidrug-resistant; G-, gram-negative bacteria; G+, gram-positive bacteria. Boldface indicates statistical significance where *p* < 0.05 (*).

**FIGURE 2:**
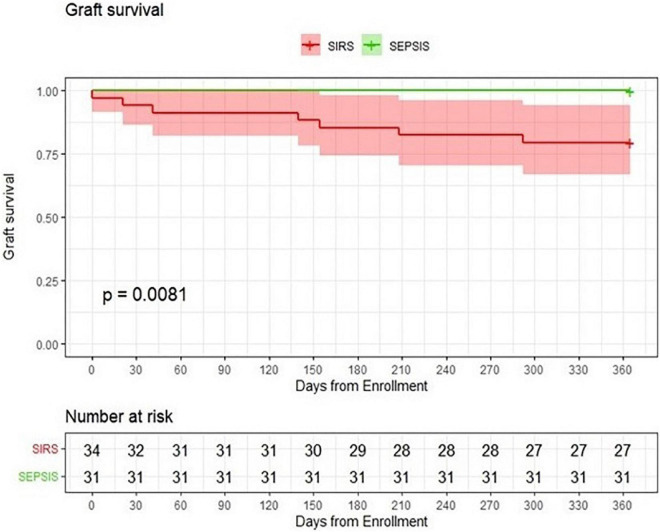
Probability of graft survival. Comparison of kidney transplant patients with sepsis with a group of kidney transplant patients with SIRS.

Finally, univariate Cox regression was used to analyze the risk factors associated with loss of kidney graft function in both patient groups together. Sepsis *per se* increased the risk of graft loss 15-fold (HR = 15.07, *p* = 0.007). Interestingly, immunosuppression without MMF (HR = 0.20, *p* = 0.038), APACHE II (HR = 1.25, *p* = 0.004), and immunosuppression based on tacrolimus (HR = 0.21, *p* = 0.041) proved significant. Graft survival was not significantly affected by any demographic factor (age, sex, and BMI), comorbidity (hypertension, diabetes, and ischemic heart disease) or immunosuppression protocol based either on cyclosporine or prednisone only (Table 5B and Figure 2).

**TABLE 5B:** Univariable Cox regression of risk factors associated with kidney graft failure one year after enrollment (*n* = 65).

Variable	HR (95% CI)	*P*-value
Immunosuppression without MMF	0.20 (0.05–0.91)	0.038*
APACHE II	1.25 (1.08–1.46)	0.004*
Age	1.04 (0.98–1.10)	0.192
Hypertension	0.87 (0.11–7.24)	0.898
Prednisone only	4.68 (0.56–38.94)	0.154
Sex (male)	4.46 (0.54–37.08)	0.166
DM I + II	1.15 (0.26–5.16)	0.852
IHD	4.13 (0.8–21.28)	0.09
Cyclosporine	4.30 (0.83–22.19)	0.081
BMI	1.01 (0.87–1.17)	0.952
Tacrolimus	0.21 (0.05–0.94)	0.041*
Sepsis patients	15.07 (1.84–1955.60)	0.007*

APACHE II, Acute Physiology and Chronic Health Evaluation II; BMI, body mass index; DM I + II, diabetes mellitus type I and type II; HR, hazard ratio; CI, confidence interval; SIRS, systemic inflammatory response syndrome; MMF, mycophenolate mofetil; IHD, ischemic heart disease. Boldface indicates statistical significance where *p* < 0.05 (*).

## Discussion

In general, sepsis is a leading cause of ICU admission, and is associated with a high mortality rate (17, 18). To determine the negative impact of sepsis on kidney graft function from a long-term perspective, we chose kidney allograft recipients with SIRS after transplantation (i.e., with similar clinical signs to sepsis, but without the presence of infection) as controls. These patients could be used as suitable controls, because any surgery (including kidney transplantation) may lead to SIRS by itself and may affect the allograft function. These patients were followed for one year after transplantation and all their grafts remained functional within this defined time period. Baseline characteristics such as demographic parameters, presence of comorbidities and immunosuppressive therapy were similar in both groups. The APACHE II score was statistically significantly higher in the group of patients with sepsis, reflecting the severity of the condition upon ICU admission.

According to our findings, sepsis significantly affected kidney graft function: 20% of patients lost graft function within one year of the septic episode and returned to dialysis. Sepsis patients also suffered from more subsequent infections and had higher one-year mortality: fungal infections, median SAPS II score, respiratory failure, and hemodynamic instability were all identified as significant one-year mortality factors. The development of septic acute kidney graft injury requiring RRT seems to be a crucial risk factor for complete loss of graft function, unlike the number of subsequent infections or duration of immunosuppression withdrawal. Notably, all recipients who required RRT due to sepsis and lost graft function died within a year of the septic event. The tacrolimus-based immunosuppression protocol was associated with loss of kidney graft functin as well as immunosuppression without MMF (Tables 5A,B). The longer time period between transplantation and enrollment in sepsis patients or selection bias may explain why these patients were placed on tacrolimus and significantly fewer MMF (as presented in Table 1).

Renal circulation plays an important role in the pathogenesis of AKI. Therefore, in instances of systemic vasodilatation, the use of vasoactive drugs is necessary in order to maintain the perfusion pressure of the kidney graft and preserve kidney function (19). However, it is also necessary to ensure sufficient intravascular volume first before titrating the appropriate dose in order to prevent further medullary hypoxia (12). This is probably why, in our study, the maximum (and not the cumulative) dose of norepinephrine proved a significant mortality factor in kidney transplant recipients with sepsis. The maximum norepinephrine dose indicates the degree of hemodynamic instability, which determines the severity of a patient’s clinical condition. On the other hand, neither the use of norepinephrine *per se* nor its maximum dose was associated with impaired or lost kidney graft function in kidney graft recipients with sepsis. In this context, it can be assumed that the fluid management and dosage of vasopressor circulatory support ensured the appropriate conditions for the preservation of kidney graft function.

In a study by the RESITRA group, crude one-year BSI-associated mortality in transplant recipients was 7.8% (5). However, in our study, 20% of sepsis patients died within one year, a difference possibly explained by variations in study design and cohort size. While the RESITRA study was multicenter in design, containing data on recipients of different solid organs as well as hematopoietic stem cells, our work was performed in a single transplant center and focused on kidney transplant recipients only.

Given that transplant patients are more vulnerable in a critical condition due to chronic immunosuppression and comorbidities, a tailored treatment approach is required. A recent retrospective multicenter study documented better results in the treatment of sepsis in immunosuppressed patients in hospitals that had a higher number of these specific patients (20). Even though the cohort of patients in this study was largely heterogeneous and transplanted patients formed only part of the cohort, it can be concluded that treatment of sepsis in transplant patients should be performed under the supervision of an experienced specialist.

Sepsis and its consequences have been the focus of many studies, but little is known about the consequences of sepsis in transplanted patients requiring long-term immunosuppression to prevent rejection (21). A retrospective multicenter cohort study, which examined in-hospital mortality of various organ transplant patients with sepsis (22), found that, contrary to expectations, in-hospital mortality was lower in transplanted than in non-transplanted patients. In this study, in-hospital mortality of transplant recipients with severe sepsis was 5.5%, whereas in non-transplanted patients it was 8.7%. In our cohort, in-hospital mortality rate was 6%. However, the comparison is not relevant given that we applied the current definition of sepsis (only sepsis, not severe sepsis according to the SSC definition). Furthermore, our cohort consisted of kidney transplant recipients only. Lower 28-day and 90-day mortality rates were also reported by another case-control study (23) in recipients of various organs (only 12.2% kidney) with bacteremic sepsis compared to non-transplanted patients. The overall 28-day mortality and 90-day mortality reported in this study was 8.1 and 14.6%, respectively. These findings may be attributed to a greater level of specialized care, a focus on detecting sepsis in these patients at an earlier stage, and the likely benefit of immunosuppression in the modulation of the inflammatory response.

Although reduction and/or withdrawal of immunosuppression is a generally accepted part of sepsis therapy in transplant recipients, data concering its impact on overall clinical outcomes and allograft function are scarce. Specifically, there is no consensus on the management of immunosuppressive drugs in critically ill patients with sepsis, nor is it fully clear whether short-term withdrawal for a necessary period of time in a sepsis setting leads to a significantly higher incidence of allograft rejection (21, 24, 25). Based on the biopsy findings in our cohort, it can be concluded that a transient reduction in immunosuppression during sepsis did not lead to the development of rejection.

In our study, we failed to demonstrate an association between temporary discontinuation of immunosuppression during sepsis and loss of kidney graft function or mortality within 1 year after sepsis.

Sepsis survivors have an increased risk of sepsis recurrence, which can be related to a compromised immune system, impaired organ function, or reduced functional reserve of the organism in response to an insult (16). Within one year after sepsis, we observed a higher incidence of hospital and community-acquired infections in kidney transplant patients with sepsis than in the control group with SIRS, even though the difference was not statistically significant. In both groups of patients, we observed a higher incidence of hospital-acquired infections than of community-acquired infections, a difference that trended toward statistical significance.

In agreement with previous findings (4, 5, 26), in our patients, the urinary tract proved the most common site as well as source of BSI, of which MDR microorganisms played a significant role.

The main strength of our study is the high homogenity of the cohort enrolled, which contained only kidney allograft recipients from a single center. Also, patients were treated by a uniform team and according to the same protocols. Sepsis was diagnosed and treated in line with recent guidelines (SSC), as was the reduction or possible withdrawal of immunosuppressive therapy. Another advantage of our study is its prospective design; most studies on sepsis in transplant patients are retrospective (22, 23, 27). The majority of previous studies have compared selected parameters in transplanted and non-transplanted patients with sepsis. In this context, our study is unusual in that it compares kidney transplant patients with sepsis and with SIRS. Our comparison of patients with sepsis and SIRS demonstrates the negative impact of infection and organ dysfunction on the prognosis of kidney recipients.

Nonetheless, our study has some limitations. Firstly, its observational design by itself. The main weakness relates to the to the small number of patients in the cohort. The study was restricted in its focus on carefully selecting patients meeting strict exclusion criteria. A major limitation in terms of recruiting patients was the condition of no antibiotic treatment within one month before enrollment, and requirement to be enrolled before the first dose of antibiotics was administered. As a result, some sepsis transplant patients meeting the other criteria were not enrolled and some degree of selection bias could arise. The control group included kidney transplant patients who were diagnosed with SIRS without infection during 30 days after transplantation.

In conclusion, our prospective single-center study confirms that sepsis in kidney transplant patients is associated with increased mortality and places them at high risk of losing kidney allograft function. However, it seems that SIRS without infection does not have negative consequences for one-year mortality and allograft function in kidney transplant patients. The requirement for renal replacement therapy in sepsis patients appears to have a particularly negative impact on the long-term function of the transplanted kidney.

## Data availability statement

The raw data supporting the conclusions of this article will be made available by the authors, without undue reservation.

## Ethics statement

The studies involving human participants were reviewed and approved by Ethics Committee of Thomayer’s Hospital and Institute for Clinical and Experimental Medicine, Prague, Czech Republic (Docket No.: G-16-06-17). The patients/participants provided their written informed consent to participate in this study.

## Author contributions

MP: patient recruitment, data acquisition, analysis and interpretation, and manuscript preparation and critical revision. EU: data analysis and interpretation and manuscript preparation and critical revision. VI and JL: patient recruitment and data collection. OV: critical revision of the manuscript. PH: data analysis and interpretation. EK: article creation, concept and design, data analysis and interpretation, and manuscript preparation and critical revision. All authors contributed to this article and approved the submitted version.
