# Supplementary material for: Sepsis affects kidney graft function and one-year mortality of the recipients in contrast with systemic inflammatory response
Source: Front Med (Lausanne). 2022 Jul 29;9:923524. doi: 10.3389/fmed.2022.923524 (PMC9372308; doi:10.3389/fmed.2022.923524)
Supplement: Supplementary file 1 [file Table_1.DOCX]

**Supplementary Table 1 |** Binary logistic regression of potential risk factors associated with impaired kidney graft function (serum creatinine above 150 μmol/l) 1- year after the study enrolment.

| \| **variables** \| **OR (95%CI)** \| \| --- \| --- \| | | \| **P-value** \| **P-value** \| \| --- \| --- \| |
| --- | --- | --- | --- | --- | --- | --- |
| APACHE II | 1.02 (0.93- 1.12) | 0.673 |
| Age | 1 (0.96- 1.04) | 0.940 |
| More than 1 event of sepsis -1 year follow up | 0.85 (0.25- 2.72) | 0.784 |
| Community acquire infection -1 year follow up | 0.97 (0.27- 3.21) | 0.962 |
| Hospital acquire infection- 1 year follow up | 1.24 (0.39- 4.03) | 0.717 |
| BMI | 1.14 (1.01- 1.3) | **0.042*** |

*APACHE II: Acute Physiology and Chronic Health Evaluation II,* *BMI: body mass index, CI: confidence interval,* OR: odds ratio. *Boldface indicates statistical significance where p< 0.05(*).*
